# Comparative gene expression profiles between heterotic and non-heterotic hybrids of tetraploid *Medicago sativa*

**DOI:** 10.1186/1471-2229-9-107

**Published:** 2009-08-13

**Authors:** Xuehui Li, Yanling Wei, Dan Nettleton, E Charles Brummer

**Affiliations:** 1 Center for Applied Genetic Technologies, University of Georgia, Athens, Georgia 30602, USA; 2 Department of Statistics, Iowa State University, Ames, Iowa 50011, USA

## Abstract

**Background:**

Heterosis, the superior performance of hybrids relative to parents, has clear agricultural value, but its genetic control is unknown. Our objective was to test the hypotheses that hybrids expressing heterosis for biomass yield would show more gene expression levels that were different from midparental values and outside the range of parental values than hybrids that do not exhibit heterosis.

**Results:**

We tested these hypotheses in three *Medicago sativa *(alfalfa) genotypes and their three hybrids, two of which expressed heterosis for biomass yield and a third that did not, using Affymetrix *M. truncatula *GeneChip arrays. Alfalfa hybridized to approximately 47% of the *M. truncatula *probe sets. Probe set signal intensities were analyzed using MicroArray Suite v.5.0 (MAS) and robust multi-array average (RMA) algorithms. Based on MAS analysis, the two heterotic hybrids performed similarly, with about 27% of genes showing differential expression among the parents and their hybrid compared to 12.5% for the non-heterotic hybrid. At a false discovery rate of 0.15, 4.7% of differentially expressed genes in hybrids (~300 genes) showed nonadditive expression compared to only 0.5% (16 genes) in the non-heterotic hybrid. Of the nonadditively expressed genes, approximately 50% showed expression levels that fell outside the parental range in heterotic hybrids, but only one of 16 showed a similar profile in the non-heterotic hybrid. Genes whose expression differed in the parents were three times more likely to show nonadditive expression than genes whose parental transcript levels were equal.

**Conclusion:**

The higher proportions of probe sets with expression level that differed from the parental midparent value and that were more extreme than either parental value in the heterotic hybrids compared to a non-heterotic hybrid were also found using RMA. We conclude that nonadditive expression of transcript levels may contribute to heterosis for biomass yield in alfalfa.

## Background

Heterosis is a phenomenon in which offspring show increased fitness relative to their parents [1]. In classic quantitative genetics, three main hypotheses have been proposed to explain heterosis [2]. One is the dominance hypothesis, which suggests heterosis results from the complementation of favorable alleles of different loci in F_1 _hybrids. Under the dominance hypothesis, each heterozygous locus in F_1 _hybrids contributes to a trait value within the range of the two homozygous parents, but summing locus effects across the genome gives the hybrid its advantage over its parents. The second is the over-dominance hypothesis, which states that a heterozygous locus in an F_1 _hybrid will perform better than either homozygous locus in parents; therefore, heterozygosity *per se *causes heterosis. Finally, the third hypothesis suggests that epistasis plays the predominant role in heterosis expression, and recent evidence in *Arabidopsis *shows that it plays a role in heterosis of biomass [3]. All three hypotheses postulate that physical allelic variation between parents results in allelic interactions at given loci in F_1 _hybrids, which in turn causes heterosis. Although not always explicitly stated, all three mechanisms concurrently may play a role in heterosis.

The underlying genetic causes of heterosis are not understood. Alleles at a given locus may be expressed at different levels [4,5], and heterosis may be explained at the molecular level by the combined allelic expression in F_1 _hybrids, and in particular, by nonadditive expression, at each locus involved in a trait [6]. Nonadditive expression in transcript levels could be classified in two ways. First, the hybrid expression level could be different from the midparental value but within the range of the parental values. Second, the hybrid expression could be outside of the parental expression level, such that the hybrid's expression is significantly above the high parent or below the low parent.

Nonadditive expression in F_1 _hybrids has been documented in several cases. In maize, Auger et al [7] used northern blot assays to analyze 30 transcripts in two maize inbred lines and their two reciprocal hybrids and found that 19 and 20 transcripts showed nonadditive expression. Of the 24 genes showing nonadditive expression in at least one hybrid, 16 showed hybrid patterns that fell outside the parental range of expression. More recent microarray experiments conducted on the same maize hybrid family (B73 × Mo17) have shown ~20% of genes show nonadditive expression [8,9]. However, these two experiments differed in the number of genes whose expression was higher or lower than the parental values, ranging from about 14% of genes [9] to nearly none [8]. Similar experiments have been conducted in Arabidopsis, Drosophila, and rice [10-13], all of which show substantial nonadditive gene expression, but the number of genes whose expression was outside the parental range is variable. However, the different degrees and types of nonadditive expression observed in these studies could be due to biological, technical, and/or statistical analysis differences, so generalizations about nonadditive gene expression in hybrids across studies and species are difficult. Unfortunately, none of these experiments assessed gene expression in hybrids that do not show a heterotic response for the trait of interest, making conclusions that nonadditive expression is related to heterosis difficult to support. More recently, an analysis of six hybrids expressing varying levels of high parent heterosis for different seedling traits found similar expression patterns among the hybrids [14]. The authors suggest that differences in transcriptional diversity among parents, rather than expression patterns *per se *in hybrids, may be involved with heterosis expression.

Cultivated *Medicago sativa *(alfalfa) is a tetrasomic tetraploid consisting of two major subspecies, *M. sativa *subsp. *sativa *and subsp. *falcata*. Hybrids between these groups often express heterosis for biomass yield and other quantitative traits [15-19]. This finding may help breeders improve the yield of this important forage crop, which has recently seen productivity plateau [18,20]. While these field-based observations demonstrate the potential for heterosis expression in alfalfa, a fuller understanding of the molecular genetic mechanisms causing heterosis could assist breeders in reliably creating high-yielding hybrids.

In this experiment, we grew three tetraploid alfalfa hybrids, two of which expressed heterosis for biomass yield in field experiments and a third that did not [18], and assessed global gene expression using Affymetrix *Medicago *GeneChip arrays. With these data, we tested the hypotheses that (i) more genes with nonadditive expression levels would be identified in heterotic than in non-heterotic hybrids when hybrids were compared to their respective parents, (ii) more genes would show expression levels that were higher than the high parent or lower than the low parent in heterotic than in non-heterotic hybrids, and (iii) the two heterotic hybrids would similar numbers of genes would show non-additive expression levels or levels of expression outside the parental range.

## Results

The signal intensities of the 24 arrays (6 entries × 4 replications) were consistent across the four replications of each individual entry as well as across all entries. No arrays were obvious outliers in terms of median or distribution of signal intensities (data not shown).

### Heterosis expression

The hybrids H12 and H13 showed significant mid-parent heterosis for biomass, while hybrid H23 did not (Table 1). The entries we used in this experiment were grown in the growth chamber, but the biomass production we measured in this experiment showed the same relative patterns of heterosis as observed previously in field experiments [18]. The low yield of WISFAL-6 is attributable to its slower regrowth compared to the two sativa parents.

**Table 1:** Dry weight for three parental alfalfa genotypes and their hybrids and the mid-parental heterosis values of the hybrids.

Entry	Dry weight	Mid-Parent Heterosis	Hybrid vs. Midparent
	g/plant		*p*-value
P1 (WISFAL-6)	0.56	--	
P2 (ABI408)	2.11	--	
P3 (C96-513)	2.57	--	
H12 (WISFAL-6 × ABI408)	2.05	0.71	0.0029
H13 (WISFAL-6 × C96-513)	2.35	0.79	0.0011
H23 (ABI408 × C96-513)	2.70	0.36	0.1295

### Probe set hybridization patterns based on MAS detection calls

Of the total 61,278 probe sets on the *Medicago *chip, 25,604 (41.8%) were 'present' in at least one of the six entries in this experiment. Of these probe sets, 71.0% were present in all entries, 20.8% were present in two to five entries, and 8.2% were unique to one entry. The 61,278 probe sets were designed from 3 species: *M. sativa*, *M. truncatula*, and *S. meliloti*. About 90.6% (1,711 of 1,888) of the probe sets derived from *M. sativa *but only 46.6% (23,700 of 50,905) of those from *M. truncatula *and 1.2% (99 of 8,305) of those from *S. meliloti *were scored as present in at least one of the six entries. Of these probe sets, 90.4%, 69.7% and 1.0%, respectively, were present in all entries and 2.0%, 8.4% and 71.7%, respectively, were present only in one single entry. Because our experimental material was *M. sativa*, the observed hybridization percentages are not surprising. The 10% of *M. sativa *genes that were not present in any individual may represent genes that were not expressed in leaves at this developmental stage and under these environmental conditions, or that were expressed at a level too low to be detected.

### Comparisons between parents

#### MAS results

**Figure 1:**
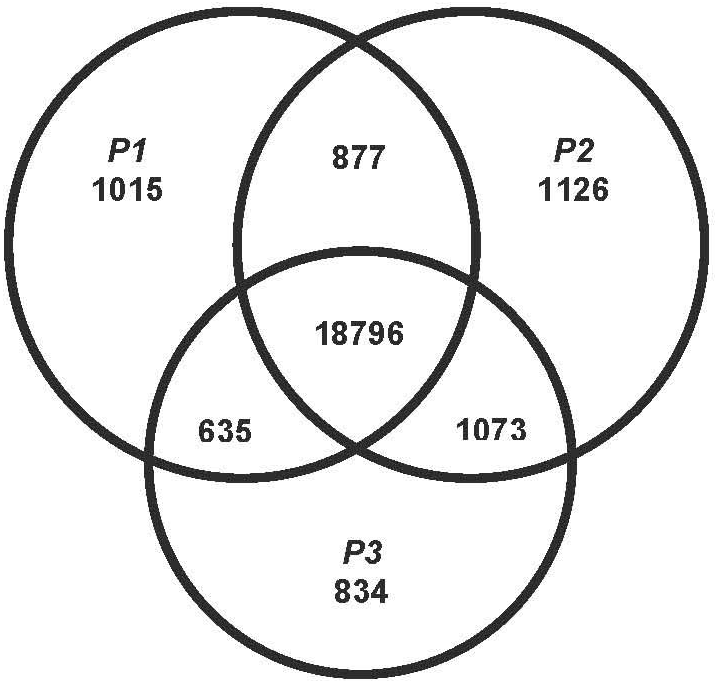
**The numbers of probe sets present in one, two, or three parental genotypes**.

Of the 24,356 probe sets that were present in at least one of the three parents, 18,796 were present in all parents and 2,975 were only present in a single parent (Figure 1). The number of probe sets present in only one parent did not differ substantially among the three parents, and P1 (WISFAL-6), which derived from *M. sativa *subsp. *falcata*, is not obviously different from the two subsp. *sativa *parents in terms of hybridization efficiency.

Of the probe sets present in at least one parent, 10,130 showed different expression levels among the three parents. For the non-heterotic parent pair P2–P3, 4,222 of 23,341 probe sets (18.1%) were found to be differentially expressed between parents, while for the heterotic parent pairs, 7,062 of 23,522 (30.0%) were differentially expressed between P1 and P2, and 7,227 of 23,230 (31.1%) between P1 and P3 (Table 2). Despite the variation among parent pairs in the number of differentially expressed genes, each parent in each pair had higher expression for about half of the probe sets (Table 2).

**Table 2:** The numbers and proportions of probe sets with significantly different expression levels between parental pairs, fold change in expression levels between parents at a false discovery rate of 0.15, and numbers of genes expressed only in one genotype of each parent pair.

Method	Parental comparison	Differentially expressed genes	Genes with higher expression in first parent of pair listed in second column		Fold change of all differentially expressed genes		Genes with >2 fold change	Genes present in one parent and absent in the other		
		no.	no.	%	minimum	median	maximum	no.	no.	%
MAS	P1 vs P2	7062	3814	54.0	1.17	1.92	711	3196	420	5.9
	P1 vs P3	7227	3608	49.9	1.16	1.95	1141	3385	480	6.6
	P2 vs P3	4222	2009	47.6	1.18	1.92	324	1960	329	7.8
RMA	P1 vs P2	12627	6752	53.5	1.04	1.41	312.3	1890	--	--
	P1 vs P3	12821	6538	51.0	1.05	1.41	180.8	2039	--	--
	P2 vs P3	8147	4028	49.4	1.03	1.40	175.6	1179	--	--

The probe sets with significantly different expression between each pair of parents had between 1.16 and 1141 fold change, with an overall median fold change of 1.93; all three parent pairs showed similar median fold change (Table 2). Considering only those probe sets having at least a 2-fold difference in expression, 1,960 probe sets displayed different expression for the non-heterotic parent pair P2–P3, compared to 3,196 and 3,385 for the heterotic parent pairs P1–P2 and P1–P3, respectively (Table 2). Of the probe sets that had different expression between parents, only about 6–8% were present in one parent and absent in the other (Table 2). This indicated that transcriptional diversity among genotypes was mainly due to transcript abundance rather than the presence or absence of expression.

#### RMA results

A total of 17,387 probe sets showed different expression levels among the three parents when analyzed with RMA. The RMA Results showed patterns similar to the MAS results. Heterotic parent pairs had more differentially expressed genes than the non-heterotic parent pair and each parent of a particular cross contributed about 50% of the genes with higher expression (Table 2). The RMA analysis identified more differentially expressed probe sets but fewer probe sets that showed fold changes greater than two when compared to MAS (Table 2). Interestingly, however, only a fraction of the probe sets identified as differentially expressed by MAS for a given parental pair were also identified by RMA as being differentially expressed for that same parental pair (P1–P2 = 23%; P1–P3 = 24%; P2–P3 = 17%).

### Comparisons between parents and their hybrid

#### MAS results

**Figure 2:**
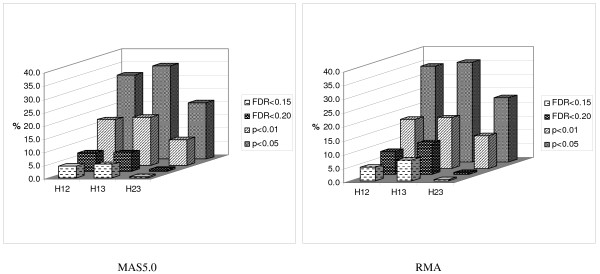
**The proportion of genes showing nonadditive expression at four statistical threshold levels for the three hybrids**. FDR is the false discovery rate.

We further analyzed each hybrid family separately to determine the proportion of probe sets showing nonadditive expression and the prevalence of hybrid expression values outside the parental range of expression. Using a cutoff of FDR < 0.15, 12.5% of probe sets displayed different expression levels among the three entries in the non-heterotic hybrid family H23, but in the heterotic hybrid families, 26.3% in H12 and 27.6% in H13 showed differences (Table 3). For each hybrid family, the probe sets with different expression can be divided into those in which the hybrid exhibits additivity of expression relative to its parents and those exhibiting nonadditive expression. We evaluated the number of probe sets with nonadditive expression using four significance thresholds (*p *< 0.05, *p *< 0.01, FDR < 0.20, and FDR < 0.15). The numbers varied dramatically among the four cutoff levels as expected, but importantly, in all cases, the heterotic hybrids (H12 and H13) showed substantially more nonadditively expressed probe sets than the non-heterotic hybrid (Figure 2).

**Table 3:** The numbers and proportions of probe sets exhibiting nonadditive expression and expression levels outside the parental range in each hybrid family at a false discovery rate of 0.15.

	MAS						RMA					
Probe set classification	Heterotic hybrids				Non-heterotic hybrid		Heterotic hybrids				Non-heterotic hybrid	
	
	H12		H13		H23		H12		H13		H23	
	
	no.	%	no.	%	no.	%	no.	%	no.	%	no.	%
Present in at least one parent or hybrid	24174	39.4	24296	39.6	23963	39.1						
Present and differentially expressed (MAS) or differentially expressed (RMA)	6346	26.3	6696	27.6	2986	12.5	11942		12015		6209	
Differentially expressed with nonadditive expression	279	4.4	334	5.0	16	0.5	591	4.9	922	7.7	34	0.5
Non-additive expression as above or below the parental range	128	45.9	156	46.7	1	6.2	329	55.7	428	46.4	14	41.2

The total number of probe sets on the GeneChip is 61,278.

We calculated the numbers of probe sets showing nonadditive expression that also had different expression levels between the parents. In all three hybrid families, a higher proportion of nonadditively expressed genes were identified in the subset of probe sets that were differentially expressed between parents than in those not differentially expressed between parents. The lower limit of the 95% confidence interval for the odds ratio under all four cutoffs was approximately three or greater (Table 4), which indicated that probe sets whose expression differed between the parents had odds of nonadditive expression that were at least three times greater than the odds of nonadditive expression for probe sets whose expression did not differ between parents. Thus, heterotic hybrids showed more nonadditive expression, and the proportion of differentially expressed probe sets in heterotic parent pairs was higher than for the non-heterotic pair.

**Table 4:** Confidence limits (95%) for the ratio of the odds of nonadditivity for probe sets that are differentially expressed between parents to the odds of nonadditivity for probe sets that are not differentially expressed between parents

Family	p < 0.05	p < 0.01	FDR < 0.20	FDR < 0.15
H12	(5.3, 6.4)	(2.9, 3.7)	(3.7, 5.5)	(4.2, 7.2)
H13	(6.5, 7.9)	(3.7, 4.8)	(4.1, 6.0)	(4.8, 7.9)
H23	(18.2, 27.5)	(7.7, 12.9)	(9.7, 170.5)	(22.6, ~)

The probe sets with nonadditive expression were divided into two categories: (i) those in which the hybrid expression level fell within the parental range of expression and (ii) those in which the hybrid expression value fell outside the parental range of expression. Greater proportions of probe sets were found to fall outside the parental range of expression in heterotic hybrids than in the non-heterotic hybrid based on FDR < 0.15 (Table 3) and also under the other three statistical thresholds (data not shown). Approximately 300 probe sets displayed nonadditive expression in each of the heterotic hybrids, and about half of these had expression levels that were higher than the higher parent or lower than the lower parent (Table 3). Of the 69 probe sets with non-additive expression that were in common between the two heterotic hybrids, 65 did not display nonadditive expression in the non-heterotic hybrid H23 (see Additional file 1). In the non-heterotic H23 hybrid family, no probe set was expressed only in the hybrid or only in both parents. In contrast, one probe set in H12 and 10 in H13 were expressed only in the hybrid (see Additional file 2).

#### RMA results

The RMA Results were similar to the MAS Results in that more probe sets with non-additive expression and with expression outside of the parental range were found in heterotic hybrid families than in non-heterotic hybrid families (Table 3 and Figure 2). However, only two and four probe sets showing non-additive expression overlapped between analysis Methods for the H12 and H13 hybrid families, respectively, and no probe sets overlapped for the H23 hybrid family, using a cutoff of FDR < 0.15. A total of 124 probe sets showed non-additive expression in both heterotic hybrids, 119 of which did not show non-additive expression in the non-heterotic hybrid H23 (see Additional file 3).

### Validation of gene expression via quantitative Real Time PCR (qRT-PCR)

**Figure 3:**
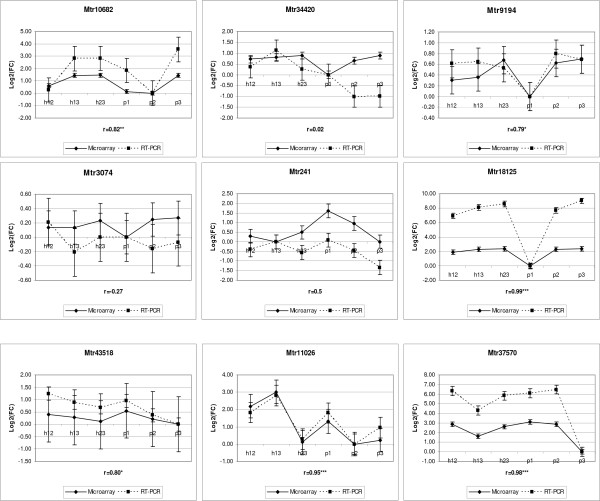
**Validation of nine probe sets using quantitative Real-Time PCR (qRT-PCR)**. The log_2_-fold change of each entry relative to the entry with the minimum expression on the microarray for each probe set is plotted for both the microarray and the qRT-PCR results. Correlations between them are shown as "r". *, ** and *** represent significance level of 0.1, 0.05 and 0.01, respectively. The standard errors are represented by the vertical bars. Note that the y-axis scale differs for each gene.

Quantitative RT-PCR was applied to 9 probe sets to verify the microarray data. Two of the probe sets, Mtr3074 and Mtr43518, did not differ among the six entries and all others showed differences in expression between at least two of the six entries based on the MAS data. In general, the qRT-PCR results produced relative expression patterns similar to those observed from the MAS analysis (Figure 3). However, some differences were evident. For Mtr34420, several entries had different expression patterns than those observed from the MAS analysis, and one entry with a different pattern than the MAS analysis was observed for Mtr241. A total of 135 pairwise comparisons for expression patterns are possible among the six entries across all nine probe sets (i.e., 15 pairwise comparisons for each probe set). Of these 135, 90 (67%) were validated by qRT-PCR. Out of 15 comparisons, only 4 and 5 were validated for probe set Mtr34420 and Mtr241, respectively, while 9 to 14 comparisons were validated for other probe sets. When compared to the RMA data, 77 (57%) of 135 pairs of comparison were validated by qRT-PCR. These results suggest that overall, the broad pattern of our microarray results is an accurate depiction of the gene expression levels among these entries.

## Discussion

A number of algorithms are available for calculating the expression intensities on Affymetrix microarrays. Among them, MAS and RMA are two of the most widely used. Comparative studies using spike-in or dilution controls have suggested that RMA algorithms are more accurate than MAS [21,22], but other experiments suggest that detection calls effectively filtered MAS data, removing the vast majority of false positive results, and that the filtered-MAS data yielded better results than RMA [23-25]. The contrasting results could be due to the different datasets, assessments, and assessment statistics used in different studies.

In this study, more differentially expressed genes between parental pairs were identified by RMA than by MAS, but smaller proportions of them showed fold changes greater than two. This supports the hypothesis proposed by previous studies [22,25] that the RMA algorithm is more sensitive, particularly at low expression levels, but this may increase the proportion of false positive results, thereby increasing noise in the data [25]. Given the conflicting results of previous experiments, we analyzed our data using both methods – MAS and RMA – to determine if the results we obtained were consistent across analysis methods.

The entries used in this study were previously tested in a field experiment [16,18], which showed that the heterotic hybrids exhibited high parent heterosis for biomass yield and that these heterotic hybrids showed greater heterosis as the period of regrowth increased. Our growth chamber results indicated that the heterotic hybrids exhibited mid-parent heterosis, probably due to the shorter length of regrowth at harvest, which we limited to three weeks to avoid possible changes in gene expression due to flowering time differences, and/or to the very different environmental conditions in the chamber compared to the field. Mid-parent heterosis for biomass may not be useful for breeding applications, but it is meaningful for the genetic study of heterosis because the difference between the hybrid and the parental mean is the response variable to be related to nonadditive expression, not their absolute phenotypic performance.

We compared two hybrids expressing heterosis for biomass yield with a third hybrid that did not express heterosis. The heterotic hybrid families had a higher number and a higher proportion of genes exhibiting differential expression and nonadditive expression than did the non-heterotic family using either analysis method (RMA or MAS). Higher proportions of probe sets with expression outside of the parental range were also found in heterotic hybrids compared to a non-heterotic hybrid. At an FDR < 0.15, we found about 300 nonadditively expressed genes in heterotic hybrids based on MAS, about half of which had expression outside the parental range, compared to 16 in the non-heterotic hybrid. Similar patterns were seen with RMA. Our data suggest that genes that have non-additive expression in the hybrid and, more importantly, that have expression levels higher than the high parent or lower than the low parent could play a role in heterosis for biomass yield.

Although the two analysis methods produced broadly similar results, different numbers of probe sets were identified as differentially expressed by the two methods and only a small proportion of these probe sets overlapped. The algorithms use different background correction, normalization, and summarization methods [26], which could explain the non-concordance between them. Further investigation is needed to determine if one algorithm more accurately identified important genes in this experiment, although based on congruence with the RT-PCR results, MAS appeared to hold a slight advantage.

Our results stand in contrast to Stupar and Springer [8] who found very little evidence for hybrid gene expression that were nonadditive or that exceeded parental levels, and different from Uzarowska et al [27] who found a large proportion of genes showing nonadditive expression (90%) and expression outside the parental range (51%) in maize. Our results are broadly similar to those of Swanson-Wagner et al [9]. However, comparisons among experiments for the percentage of nonadditively expressed genes need to be made cautiously for a number of reasons, including the use of different statistical methods and thresholds. Recently, a few studies compared the expression profiles of a set of hybrids simultaneously. Stupar et al [14] investigated the gene expression profile of six maize inbred-hybrid combinations with varying levels of better parent heterosis on five traits, and found a strong correlation between the number of differentially expressed genes and the level of genetic distance between inbred parents, while the proportions of nonadditive expression among the differentially expressed genes were similar among the hybrids. Interestingly, the hybrid with the smallest genetic distance – and the least high-parent heterosis for seedling traits – exhibited the greatest proportion of nonadditive expression. The authors proposed that nonadditive expression is not correlated with heterosis levels. Guo et al [28] found that heterosis was correlated with the proportion of additively expressed genes but not with the proportion of genes with expression levels outside of the parental range in a set of 16 maize hybrids.

Our study only analyzed three hybrids, limiting our ability to generalize these results to other hybrids. Perhaps more importantly, our results need to be interpreted cautiously given that we used non-inbred parents. Unfortunately, alfalfa suffers severe inbreeding depression, and true inbred lines are not available. To account for the heterogeneity of F_1 _hybrid indivduals, we pooled ten individuals for each hybrid. This can potentially lead to erroneous results, if alleles from the heterozygous parents are not present in the progeny in equal frequencies. In this case, the hybrid expression relative to the parental mean may be skewed – for example, if the progeny only received a highly expressing allele from one parent, then the overall hybrid expression level may be equal to or exceed the higher parent, even though the hybrid expression level should be additive. Without evaluating allele-specific expression patterns, this concern is difficult to allay. We examined the heterozygosity of the parents using 41 EST-SSR markers. WISFAL-6 (P1) had 1.92 alleles/marker, ABI408 (P2) had 1.95, and C96-513 had 2.15. Assuming that the SSR allele diversity mirrors the diversity of alleles producing different expression patterns, these results suggest that the three parents would have a similar chance to generate false expression results due to preferential allele inheritance. Therefore, we suggest that our comparisons among the three hybrids regarding the about the number and proportion of genes showing nonadditive expression are valid.

Although higher proportions of the nonadditive expression and expression higher or lower than either parent were found in heterotic hybrids compared to a non-heterotic hybrid in our study, the majority of genes showed additive expression in all hybrid families. We may have underestimated the numbers of genes with nonadditive expression due to limitations in our statistical power for this experiment. However, in maize, although the F_1 _hybrid between Mo17 and B73 showed significant high parent heterosis for seedling growth, only 22% of differentially expressed genes had nonadditive expression and only a small proportion of them showed expression outside of the parental range, similar to our results [9]. Springer and Stupar [29] proposed that heterosis could result from the additive expression of multiple genes, whereby particularly low or high expression values that are generally detrimental to the plant are modulated in the hybrid, which expresses an average expression level in a moderate, but more biologically functional range. While this may be true in some cases, the clear differences in expression patterns between hybrid types in our experiment suggests that nonadditive expression may also be important for heterosis expression.

What is heterosis? Heterosis simply represents the manifestation of a phenotype in a hybrid that is different from the expectation of a parental average value for that phenotype, be it yield, plant height, or any other trait. The manifestation of the phenotype – particularly of quantitatively inherited traits like yield – results from the complex actions of many components, including the timing of the expression of various genes, the magnitude and location of their expression, and the interaction of their gene products. The genetic hypothesis for the cause of heterosis that has the most empirical support at the current time is that each parent contains a set of dominant alleles at loci controlling the trait and that at some loci, the other parent has recessive alleles at those loci; thus, hybridization brings these dominant alleles together, with the parents complementing each other and giving the hybrid a larger set of dominant (and desirable) alleles than either parent. Complementary expression patterns – each parent contributing alleles that show higher expression than those at the relevant loci in the other parent – could have the same effect. Under this model, hybrids expressing heterosis should have more nonadditive expression, as we have shown in our alfalfa example. Given that control of complex traits likely involves many genes and given that the expression level of most genes is additive, this model does not exclude the possibility that additivity also plays a role in heterosis, under the model suggested by Springer and Stupar [29].

Conceivably, only a subset of genes may need to deviate from additivity of expression in order to produce a heterotic phenotype. The extent of nonadditive expression at different development stages and different tissues may vary and across the life cycle of the plant, the expression patterns cumulatively produce the observed heterotic response. *Arabidopsis *allotetraploids had little overlap between the set of genes exhibiting nonadditive expression in leaves and that in flowers, suggesting a role of developmental stages and tissue types on nonadditive gene regulation [13]. If nonadditively expressed genes truly do underlie heterosis, this result suggests that different genes contribute to heterosis in different tissues and at different developmental stages. Thus, for integrative phenotypes like yield, the cumulative effect of these different genes acting at different places and times could result in heterosis. If this is the case, then the nonadditive expression observed at a single timepoint and in a single tissue, as we assayed here, would only give a small part of the overall picture of how gene expression may affect the ultimate expression of the yield phenotype. Finally, genetic divergence between the parental lines appears to result in more differential expression between parents. Both in our study and in that in *Arabidopsis *by [13], a higher proportion of nonadditive expression occurred in hybrids whose parents showed divergent expression levels than in hybrids whose parents had similar expression levels. This suggests that there could be more nonadditive expression in the crosses between more distantly related parents, exactly the type of situation in which agronomically useful heterosis levels are also commonly observed. However, recent results in maize suggest that this may not be the case [14].

The expression levels of individual genes are themselves controlled by other genes, acting in *cis *or *trans *[8,30]. Thus, heterosis for an ultimate phenotype, in this case, biomass yield, must be controlled by multiple genes exhibiting some level of dominance, with some residing in each parental genome [2]. The genes themselves may also be controlled by a number of other genes, and this control can result in expression levels ranging from additivity to some level of non-additivity. Genes controlling transcript levels have been inferred from experiments mapping eQTL, that is, quantitative trait loci that control the expression of a transcript [5,30,31]. Interestingly, no eQTL could be mapped for some genes with highly heritable transcript levels in yeast, suggesting that many loci of small effect and/or epistasis among loci controls their expression [31].

We know that biomass yield, like many other agronomically important traits, is quantitatively inherited, suggesting that it is controlled by many loci (and possibly by multiple interactions among them), and infer that directional dominance plays a role in its control, at least in the certain hybrids that express heterosis. As a means of understanding the nature of the genetic mechanisms underlying biomass yield and yield heterosis, we identified a suite of genes whose expression in hybrids is phenotypically nonadditive, in some cases falling outside of the parental range, and a subset of which only show that expression pattern in heterotic hybrids. But expression of each individual gene is itself the result of a number of gene interactions, and hence, the regulation of expression of any single gene may also have a complex genetic basis. This complexity shows that the genetic control of quantitative traits is difficult to untangle because many levels of interactions, from genes to gene expression profiles to proteins and metabolites, occur to produce the ultimate phenotype.

## Conclusion

Gene expression profiles between two heterotic hybrids and one non-heterotic hybrid have been compared. We found that the heterotic hybrid families had a higher number and a higher proportion of genes exhibiting nonadditive expression and expression levels outside the parental range than did the non-heterotic family. We concluded that nonadditive expression and expression higher or lower than either parent might contribute to heterosis for biomass yield. However, further research is needed in order to clearly associate non-additive gene expression with heterosis for biomass yield.

## Methods

### Plant Growth, Experiment Design and Sampling

We focused on three genotypes and their hybrids. The parents consisted of one genotype from a semi-improved germplasm of subsp. *falcata*, WISFAL-6 (P1), and two elite genotypes from commercial alfalfa breeding germplasm of subsp. *sativa*, ABI408 (P2) and C96-513 (P3). These three genotypes and their hybrids (H12, H13 and H23) have been extensively evaluated for biomass yield, nutritive value, and agronomic traits in a series of previous papers [16,18,19]. The two sativa × falcata hybrids had previously exhibited heterosis for biomass yield and the sativa × sativa hybrid did not when evaluated in a field experiment [18]. For convenience in the following narrative, we refer to the three parents and their three hybrid populations as the six entries evaluated in the study. Also, we will refer to the hybrids expressing heterosis for biomass as "heterotic hybrids" and the hybrid which did not as a "non-heterotic hybrid."

The experimental design was a randomized complete block design (RCBD) with four replications. Each replication included 2 clones for each parent and a single clone for each of 10 genotypes in each hybrid family, for a total of 36 plants. Because the parents were not inbred lines, a cross between them results in a segregating F_1 _population. Thus, the ten F_1_individuals per family represented the hybrid population for the array experiment. Plants were grown in growth chambers (two replications in each of two chambers) under controlled conditions of 25°C and a 16 hr photoperiod. After being placed into the chambers, plants were maintained for 30 days at which point all biomass was clipped to a 5 cm height above soil. Twenty-three days following clipping, the upper fully expanded leaf on a given stem was sampled for RNA isolation and microarray analysis. We sampled five trifoliate leaves from each of the two clones for each parent, and one trifoliate leaf from each of 10 genotypes for each hybrid. The leaves for each parent or hybrid were pooled prior to RNA extraction. Leaves were harvested, quickly frozen in liquid nitrogen, and stored at -80°C until RNA isolation. After sampling leaves, the whole plants were cut and dried at 60°C for four days to measure the dry weight. Mid-parent heterosis for yield was calculated on a dry weight basis as the difference between the mean value of an F_1 _population and the mean of the parents.

### RNA isolation and hybridization

The total RNA for array hybridizations was extracted from frozen leaf tissue with Trizol reagent using standard procedures [32]. Gene expression was assayed using *Medicago *Affymetrix GeneChips, which include 61,278 genes identified from EST collections and genome sequencing data in *M. truncatula*, *Sinorhizobium meliloti *and *M. sativa*, together with hybridization controls, housekeeping controls, and Poly-A controls. For the experiment, four biological replications of the six entries resulted in 24 GeneChip hybridizations.

First strand cDNA synthesis, GeneChip hybridization, and array staining were conducted at the Iowa State University GeneChip Facility . Arrays were scanned with a GeneChip Scanner 3000 7G. The gene expression of each probe set on the array was determined from the scanned signal intensities using the Affymetrix^® ^MicroArray Suite v.5.0 (MAS) software and the robust multi-array average (RMA) software [22]. The data resulting from both methods have been uploaded to the MIAMExpress public database ("", accession number: E-MEXP-1579).

### Statistical analysis of microarray data

MAS determines the actual expression intensity of each probe set and provides a detection call indicating whether the estimated expression level is reliable by classifying each probe set on each chip as present (P), marginal (M), or absent (A). Thus, using MAS, we first compared genotypes based on detection calls, and second based on the actual expression intensities of each probe set, filtered by detection call as suggested by previous studies [23,24]. With RMA, we compared genotypes based on expression intensities of each probe set, the only result RMA provides.

#### Comparisons based on detection calls

Each chip contains 61,278 probe sets. Because our experiment included four replications (corresponding to four separate chips for each entry), each entry received four signal calls for each probe set. For a given entry, a probe set that was PPPP, PPPM, PPPA, or PPMM across the four replications was designated as present, a probe set that was MAAA or AAAA was designated as absent, and the remaining probe sets were designated as marginal.

#### Comparisons based on expression level differences

Expression intensity data from MAS were log transformed and normalized by median centering prior to analysis. Using the transformed and normalized MAS data and the RMA expression intensity data, we fit the following mixed linear model to each probe set:



where *μ *is the overall probe set mean, *G*_*i *_(*i *= 1,...,6) is the effect of the *i*th entry, *r*_*j *_(*j *= 1,...,4) is the effect of the *j*th replication, and *e*_*ij *_is the random error associated with the *i*th entry in the *j*th replication; *r*_*j *_and *e*_*ij *_were modeled as independent normal random effects, and the others were modeled as fixed effects.

Differential expression was evaluated (i) among the three parental entries, (ii) between the two parents of a given hybrid, and (iii) between the two parents and their hybrid by testing the null hypothesis that the entries had equal expression levels. To control for multiple testing errors, the false discovery rate (FDR) of Benjamini and Hochberg [33] was employed at a significance level of α = 0.15, as has been used in other studies of this type [9]. For MAS data, only probe sets that were identified as being present in at least one of the entries being compared were evaluated.

For each hybrid family (i.e., the two parents and their hybrid), probe sets with nonadditive expression were identified within the differentially expressed probe sets by contrasting the expression levels of the hybrid with the mean of the two parents. We were interested in whether the numbers of genes with nonadditive expression differed between heterotic and non-heterotic hybrid families. Therefore, we assessed four different significance level thresholds to determine the stability of the relationship between hybrid types, including *p*-values of 0.05 and 0.01 and FDR levels of 0.20 and 0.15. In order to test whether nonadditive expression in the hybrid tended to occur for probe sets that were differentially expressed between parents, we calculated an odds ratio (OR) to compare the number of nonadditively expressed probe sets that showed differential expression between parents and those that did not as follows:



where, *m1 *is the number of probe sets with nonadditive expression that also showed different expression levels between parents, *n1 *is the total number of probe sets whose expression was significantly different between parents, *m2 *is the number of probe sets with nonadditive expression whose expression was not significantly different between parents, and *n2 *is the total number of probe sets whose expression was not significantly different between parents. The 95% confidence limits of the odds ratio were calculated using the EXACT statement and OR option in the SAS procedure FREQ [34].

The probe sets that showed nonadditive expression were classified as being (1) outside the parental range of expression (i.e., higher than the high parent or lower than the low parent at a *p*-value of 0.05) or (2) within the parental range of expression (i.e., equal to or less than the higher parent but greater than the midparental value or equal to or greater than the lower parent but less than the midparental value at a *p*-value of 0.05).

For MAS data, we also identified probe sets that were only expressed in the hybrid in each hybrid family (i.e., the detection call was 'present' in the hybrid and 'absent' in both parents and the actual expression level was different between the hybrid and either parent at FDR < 0.15) and those expressed only in both parents and not the hybrid, using the same parameters.

### Validation of gene expression via quantitative Real-Time PCR (qRT-PCR)

In order to confirm gene expression levels detected on the Affymetrix array, we conducted qRT-PCR for nine probe sets. We selected these probe sets to represent a diversity of expression profiles among the six entries. Two probe sets (Mtr3074 and Mtr43518) did not differ among the six entries; the other seven probe sets showed differences in expression levels between at least two entries. The qRT-PCR analysis was performed on first strand cDNA synthesized from the same RNA samples used for the microarray experiment. A poly dT primer and SuperScript II RNase H Reverse Transcriptase (Cat. No. 18064-014, Invitrogen, CA) were used to synthesize first strand cDNA. Amplification primers (see Additional file 4) were designed using Primer 3 [35] for nine probe sets having contrasting expression patterns among the 6 entries based on MAS data. The qRT-PCR was conducted using first strand cDNA diluted 60 times on a LightCycler 480 SYBR Green I Master (Roche Cat. No. 04-707-516-001) following the manufacturer's protocol. The qRT-PCR data were initially analyzed with the LightCycler 480 analysis software to obtain crossing point (Cp) values for each probe set.

## Authors' contributions

XL participated in experimental design, conducted the bulk of the experimental work, analyzed the microarray and qRT-PCR data, and drafted the manuscript. YL performed qRT-PCR experiment and gene ontology analysis. DN provided advice on statistical analysis and data explanation. CB conceived, designed and supervised the study. All authors have read and approved the final manuscript.

## Supplementary Material

Additional file 1 **The putative identity of probe sets that displayed nonadditive expression in both heterotic hybrids and not in the non-heterotic hybrid based on MAS data**. The putative gene function was first cited from Affymetrix annotation if available for the probe set of interest. If not, the probe set target sequence provided by Affymetrix was used for BLASTn, BLASTx and tBLASTx search against GenBank NR with expected value lower than 1e-10. Click here for file

Additional file 2 **Probe sets and their putative identity that were only expressed in hybrids and not parents based on MAS data**. The putative gene function information was obtained as explained in Additional file 1. Click here for file

Additional file 3 **The putative identity of probe sets that displayed nonadditive expression in both heterotic hybrids and not in the non-heterotic hybrid based on RMA data**. The putative gene function information was obtained as explained in Additional file 1. Click here for file

Additional file 4 **Primers used for qRT-PCR confirmation of microarray results** Click here for file
