# Supplementary material for: Comparative gene expression profiles between heterotic and non-heterotic hybrids of tetraploid Medicago sativa
Source: BMC Plant Biol. 2009 Aug 13;9:107. doi: 10.1186/1471-2229-9-107 (PMC2736959; doi:10.1186/1471-2229-9-107)
Supplement: Additional file 1 — The putative identity of probe sets that displayed nonadditive expression in both heterotic hybrids and not in the non-heterotic hybrid based on MAS data. The putative gene function was first cited from Affymetrix annotation if available for the probe set of interest. If not, the probe set target sequence provided by Affymetrix was used for BLASTn, BLASTx and tBLASTx search against GenBank NR with expected value lower than 1e-10. [file 1471-2229-9-107-S1.doc]

### Additional file 1 - The putative identity of probe sets that displayed nonadditive expression in both heterotic hybrids and not in the non-heterotic hybrid based on MAS data

### The putative gene function was first cited from Affymetrix annotation if available for the probe set of interest. If not, the probe set target sequence provided by Affymetrix was used for BLASTn, BLASTx and tBLASTx search against GenBank NR with expected value lower than 1e-10.

| Probe ID from Affymetrix GeneChip | Putative identity | Source |
| --- | --- | --- |
| Msa.1091.1.S1_at | Glutathione S-transferase | BLAST |
| Msa.1237.1.S1_s_at | Glutathione S-transferase | BLAST |
| Msa.1368.1.S1_at | Pyruvate kinase | BLAST |
| Msa.1436.1.S1_at | Glutathione S-transferase | BLAST |
| Msa.1502.1.S1_at | Ubiquitin fusion protein or ubiquitin/ribosomal protein | BLAST |
| Msa.1865.1.S1_at | Ripening-related protein; Invertase/pectin methylesterase inhibitor family protein | BLAST |
| Msa.1924.1.S1_at | RNA-dependent RNA polymerase | BLAST |
| Msa.2667.1.S1_at | Glucosyltransferase | BLAST |
| Msa.2897.1.S1_s_at | Protein phosphatase | BLAST |
| Msa.2904.1.S1_at | Microtubule-associated protein | BLAST |
| Msa.2983.1.S1_at | Glutathione S-transferase | BLAST |
| Msa.3042.1.S1_at | BURP | BLAST |
| Msa.3081.1.S1_at | Unknown |  |
| Mtr.10348.1.S1_at | Molybdenum cofactor sulfurase protein-like | Affymetrix |
| Mtr.10595.1.S1_s_at | Seed maturation protein PM36 | Affymetrix |
| Mtr.10695.1.S1_s_at | Unknown |  |
| Mtr.10828.1.S1_at | Glucosyltransferase-13 | Affymetrix |
| Mtr.11026.1.S1_at | Unknown |  |
| Mtr.11111.1.S1_at | Dehydration-induced protein ERD15 | Affymetrix |
| Mtr.1201.1.S1_s_at | Unknown |  |
| Mtr.12333.1.S1_a_at | Unknown |  |
| Mtr.12883.1.S1_at | Unknown |  |
| Mtr.14645.1.S1_s_at | Unknown |  |
| Mtr.15870.1.S1_at | Heavy metal transport detoxification protein | Affymetrix |
| Mtr.18125.1.S1_at | Histone | Affymetrix |
| Mtr.20242.1.S1_s_at | Unknown |  |
| Mtr.23497.1.S1_at | F-box family protein | BLAST |
| Mtr.241.1.S1_at | Delta-6-desaturase | Affymetrix |
| Mtr.25749.1.S1_at | Copper amine oxidase -related | Affymetrix |
| Mtr.28547.1.S1_s_at | Glutathione S-transferase | Affymetrix |
| Mtr.30128.1.S1_at | Unknown |  |
| Mtr.31209.1.S1_s_at | Light-inducible protein | Affymetrix |
| Mtr.3122.1.S1_at | Unknown |  |
| Mtr.33526.1.S1_at | Zinc finger protein FZF | Affymetrix |
| Mtr.34420.1.S1_at | Similar to At4g32450, a pentatricopeptide repeat (PPR) -containing protein | Affymetrix |
| Mtr.35862.1.S1_at | Mitochondrial import receptor subunit TOM22 homolog | Affymetrix |
| Mtr.35929.1.S1_at | Similar to At3g57000, a nucleolar essential protein-related | Affymetrix |
| Mtr.37340.1.S1_at | Mannitol dehydrogenase (NAD-dependent mannitol dehydrogenase) | Affymetrix |
| Mtr.37380.1.S1_at | Ubiquitin-like protein | Affymetrix |
| Mtr.40279.1.S1_at | Glutathione S-transferase | Affymetrix |
| Mtr.40616.1.S1_at | Glutathione S-transferase | Affymetrix |
| Mtr.42373.1.S1_at | Peroxidase 40 precursor | Affymetrix |
| Mtr.42861.1.S1_at | Ubiquitin ribosomal protein | Affymetrix |
| Mtr.42861.1.S1_s_at | Ubiquitin ribosomal protein | Affymetrix |
| Mtr.43124.1.S1_at | Homeodomain leucine zipper protein | Affymetrix |
| Mtr.43409.1.S1_at | Unknown |  |
| Mtr.43623.1.S1_at | Zinc finger (C3HC4-type RING finger) family protein | Affymetrix |
| Mtr.43664.1.S1_at | Carboxylesterase-like protein | Affymetrix |
| Mtr.44928.1.S1_at | Similar to At1g74380, a putative galactosyl transferase | Affymetrix |
| Mtr.47086.1.S1_at | Putative Glucan 1,3-beta-glucosidase | Affymetrix |
| Mtr.47127.1.S1_at | Putative far-red impaired response protein | Affymetrix |
| Mtr.47975.1.S1_s_at | Unknown |  |
| Mtr.48425.1.S1_at | Pectinesterase family similar to pectinesterase precursor | Affymetrix |
| Mtr.50307.1.S1_at | Haloacid dehalogenase-like hydrolase; HAD-superfamily hydrolase, subfamily IA, variant 1 | Affymetrix |
| Mtr.51040.1.S1_s_at | Cytochrome b-245, heavy chain; Phenol hydroxylase reductase; or Ferric reductase-like transmembrane component | Affymetrix |
| Mtr.52076.1.S1_at | Hypoxia induced protein | Affymetrix |
| Mtr.5449.1.S1_at | N protein | Affymetrix |
| Mtr.6808.1.S1_at | GCN5-like protein | Affymetrix |
| Mtr.7160.1.S1_at | MRP-like ABC transporter | Affymetrix |
| Mtr.7832.1.S1_at | Unknown |  |
| Mtr.8445.1.S1_s_at | Extensin | Affymetrix |
| Mtr.9766.1.S1_at | Unknown |  |
| AFFX-Msa-gsta-5_x_at | Glutathione S-transferase | Affymetrix |
| AFFX-Msa-gsta-M_x_at | Glutathione S-transferase | Affymetrix |
| AFFX-Msa-gsta-3_at | Glutathione S-transferase | Affymetrix |
