# Supplementary material for: Comparative gene expression profiles between heterotic and non-heterotic hybrids of tetraploid Medicago sativa
Source: BMC Plant Biol. 2009 Aug 13;9:107. doi: 10.1186/1471-2229-9-107 (PMC2736959; doi:10.1186/1471-2229-9-107)
Supplement: Additional file 2 — Probe sets and their putative identity that were only expressed in hybrids and not parents based on MAS data. The putative gene function information was obtained as explained in Additional file 1. [file 1471-2229-9-107-S2.doc]

### Additional file 2 - Probe sets and their putative identity that were only expressed in hybrids and not parents based on MAS data

### The putative gene function information was obtained as explained in Additional file 1.

| Probe ID from Affymetryx GeneChip | Putative identity | Source |
| --- | --- | --- |
| Mtr.52167.1.S1_s_at | Immunoglobulin major histocompatibility complex | Affymetrix |
| Mtr.38224.1.S1_at | Unknown |  |
| Mtr.15572.1.S1_at | Unknown |  |
| Mtr.10565.1.S1_at | Similar to At2g46330, a putative arabinogalactan protein | Affymetrix |
| Mtr.42017.1.S1_at | Type IIB calcium ATPase | Affymetrix |
| Mtr.19620.1.S1_at | Protein kinase | Affymetrix |
| Mtr.25810.1.S1_at | Nucleic acid-binding protein | Affymetrix |
| Msa.376.1.S1_at | Unknown |  |
| Msa.2546.1.S1_at | Unknown |  |
| Msa.1627.1.S1_at | 3-oxoacyl-ACP synthase; Beta-ketoacyl-ACP synthase; 3-ketoacyl-ACP synthase | BLAST |
| Mtr.34731.1.S1_at | Viral replicase; Methyltransferase/helicase; Polymerase | BLAST |
