# Supplementary material for: Comparative gene expression profiles between heterotic and non-heterotic hybrids of tetraploid Medicago sativa
Source: BMC Plant Biol. 2009 Aug 13;9:107. doi: 10.1186/1471-2229-9-107 (PMC2736959; doi:10.1186/1471-2229-9-107)
Supplement: Additional file 3 — The putative identity of probe sets that displayed nonadditive expression in both heterotic hybrids and not in the non-heterotic hybrid based on RMA data. The putative gene function information was obtained as explained in Additional file 1. [file 1471-2229-9-107-S3.doc]

### Additional file 3 - The putative identity of probe sets that displayed nonadditive expression in both heterotic hybrids and not in the non-heterotic hybrid based on RMA data

### The putative gene function information was obtained as explained in Additional file 1.

| Probe ID from Affymetryx GeneChip | Putative identity | Source |
| --- | --- | --- |
| Mtr.23965.1.S1_s_at | Wound-inducible protein | Affymetrix |
| Mtr.18180.1.S1_at | Hypothetical protein | Affymetrix |
| Mtr.14845.1.S1_s_at | NUDIX hydrolase | Affymetrix |
| Mtr.37537.1.S1_s_at | Disease resistance protein-like protein MsR1 | Affymetrix |
| Mtr.50996.1.S1_at | Disease resistance protein; TIR; NB-ARC; Leucine-rich repeat; AAA ATPase | Affymetrix |
| Mtr.17810.1.S1_s_at | Leucine-rich repeat; Leucine-rich repeat, typical subtype | Affymetrix |
| Mtr.16090.1.S1_x_at | Hypothetical protein | Affymetrix |
| Mtr.822.1.S1_x_at | Calcineurin-like phosphoesterase family, contains Pfam profile | Affymetrix |
| Mtr.23952.1.S1_at | IMP dehydrogenase | BLAST |
| Mtr.21104.1.S1_at | Hypothetical protein | Affymetrix |
| Mtr.18204.1.S1_at | Translation initiation factor IF5; eIF4-gamma/eIF5/eIF2-epsilon | Affymetrix |
| Mtr.48849.1.S1_at | Molybdopterin cofactor biosynthesis protein MoaC | Affymetrix |
| Mtr.12291.1.S1_s_at | Proline dehydrogenase | Affymetrix |
| Mtr.12650.1.S1_at | Phosphatidylinositol transfer-like protein III | Affymetrix |
| Mtr.13413.1.S1_at | Transporter like protein | Affymetrix |
| Mtr.13584.1.S1_at | Latex cyanogenic beta glucosidase | Affymetrix |
| Mtr.14756.1.S1_at | Galactose mutarotase-like | Affymetrix |
| Mtr.15927.1.S1_at | Zn-finger, DHHC type | Affymetrix |
| Mtr.16054.1.S1_at | Nucleic acid-binding OB-fold; tRNA synthetase, class II (D, K and N) | Affymetrix |
| Mtr.17234.1.S1_at | Zn-finger; Zn-finger, PMZ type | Affymetrix |
| Mtr.18801.1.S1_x_at | Pentatricopeptide repeat; Protein prenyltransferase | Affymetrix |
| Mtr.1948.1.S1_at | NPH3 family protein | Affymetrix |
| Mtr.2040.1.S1_at | Laccase-like protein | Affymetrix |
| Mtr.22858.1.S1_at | Esterase/lipase/thioesterase family | Affymetrix |
| Mtr.23153.1.S1_at | Prolyl 4-hydroxylase | Affymetrix |
| Mtr.29238.1.S1_at | RAN binding protein 16-like | Affymetrix |
| Mtr.30489.1.S1_at | Tetrathionate reductase subunit C | Affymetrix |
| Mtr.31088.1.S1_at | Pelota-like protein | Affymetrix |
| Mtr.32297.1.S1_at | Oxidosqualene cyclase | Affymetrix |
| Mtr.33189.1.S1_s_at | AML1, RNA binding protein | Affymetrix |
| Mtr.34009.1.S1_at | Ras-related protein Rab11C | Affymetrix |
| Mtr.34468.1.S1_at | Pyridoxal kinase-like protein SOS4 | Affymetrix |
| Mtr.34524.1.S1_at | Fused-ccdB protein | Affymetrix |
| Mtr.34718.1.S1_s_at | Nicotinamide-nucleotide adenylyltransferase-like | Affymetrix |
| Mtr.34881.1.S1_at | NCOR isoform b | Affymetrix |
| Mtr.35186.1.S1_at | Major surface like glycoprotein | Affymetrix |
| Mtr.35806.1.S1_at | Isoprenylated protein FP6 | Affymetrix |
| Mtr.37528.1.S1_at | Vacuolar ATPase subunit E | Affymetrix |
| Mtr.40234.1.S1_at | Translational elongation factor 1 subunit Bbeta | Affymetrix |
| Mtr.40242.1.S1_at | Mitochondrial processing peptidase alpha subunit, mitochondrial precursor (Alpha-MPP) (Ubiquinol-cytochrome-c reductase subunit II) | Affymetrix |
| Mtr.4073.1.S1_s_at | Phosphatidylinositol 4-kinase | Affymetrix |
| Mtr.41681.1.S1_at | Phosphoglycerate kinase | Affymetrix |
| Mtr.42870.1.S1_at | 60S acidic ribosomal protein P0 | Affymetrix |
| Mtr.43226.1.S1_at | 60S ribosomal protein L30 | Affymetrix |
| Mtr.43299.1.S1_at | Progesterone-binding protein-like | Affymetrix |
| Mtr.45072.1.S1_at | Limonoid UDP-glucosyltransferase (Limonoid glucosyltransferase) (Limonoid GTase) (LGTase) | Affymetrix |
| Mtr.4887.1.S1_at | Cysteine protease 1 | Affymetrix |
| Mtr.4973.1.S1_s_at | N-acetylornithine deacetylase-like protein | Affymetrix |
| Mtr.50553.1.S1_x_at | Immunoglobulin/major histocompatibility complex | Affymetrix |
| Mtr.5479.1.S1_at | Lymphocyte specific helicase (Proliferation associated SNF2-like protein) | Affymetrix |
| Mtr.6399.1.S1_at | Pentatricopeptide (PPR) repeat-containing protein / CBS domain-containing protein-like | Affymetrix |
| Mtr.7034.1.S1_at | NADH dehydrogenase subunit 3 | Affymetrix |
| Mtr.7135.1.S1_at | Low temperature-responsive RNA-binding protein | Affymetrix |
| Mtr.7539.1.S1_at | Mitochondrial ribosomal protein S19 | Affymetrix |
| Mtr.7553.1.S1_at | Alanine acetyl transferase-like protein | Affymetrix |
| Mtr.8050.1.S1_at | All-trans-13,14-dihydroretinol saturase | Affymetrix |
| Mtr.8319.1.S1_at | Class III peroxidase 5 precursor | Affymetrix |
| Mtr.9058.1.S1_at | DNA-binding protein | Affymetrix |
| Mtr.9582.1.S1_at | Glycogen(starch) synthase isoform II precursor | Affymetrix |
| Sme.3470.1.S1_at | Uncharacterized ACR, YneC family COG1359 subfamily | Affymetrix |
| AFFX-r2-Ec-bioB-5_at | Biotin synthetase | Affymetrix |
| Mtr.41998.1.S1_s_at | Ubiquitin thiolesterase/ zinc ion binding | BLAST |
| Msa.1151.1.S1_at | Calcium-binding protein | BLAST |
| Msa.1716.1.S1_at | Zinc dependent protease; FtsH-like protein | BLAST |
| Msa.414.1.S1_at | Unknown |  |
| Msa.2978.1.S1_at | Chlorophyll a/b binding protein (CARCAB1) | BLAST |
| Sme.5329.1.S1_at | Hypothetical protein | Affymetrix |
| Msa.1914.1.S1_at | Ribosomal protein S26 | BLAST |
| Msa.2356.1.S1_at | Unknown |  |
| Mtr.7064.1.S1_at | NEC1 | Affymetrix |
| Mtr.6092.1.S1_at | Protein transporter | BLAST |
| Mtr.11116.1.S1_at | Glycosyl transferase | BLAST |
| Mtr.16659.1.S1_s_at | H+transporting two-sector ATPase, alpha/beta subunit, central region | BLAST |
| Mtr.19572.1.S1_at | Unknown | BLAST |
| Mtr.19945.1.S1_at | Quercetin 3-O-glucoside-6''-O-malonyltransferase; Anthocyanin 5-aromatic acyltransferase (5AT) | BLAST |
| Mtr.21227.1.S1_s_at | Hypothetical protein | Affymetrix |
| Mtr.18730.1.S1_at | Reverse transcriptase | BLAST |
| Mtr.24387.1.S1_at | Unknown |  |
| Mtr.25962.1.S1_at | Unknown |  |
| Mtr.30596.1.S1_at | Unknown |  |
| Mtr.10120.1.S1_at | Unknown |  |
| Mtr.11521.1.S1_at | Tetrachloro-p-hydroquinone reductive dehalogenase | BLAST |
| Mtr.2159.1.S1_at | Unknown |  |
| Mtr.25717.1.S1_at | Unknown |  |
| Mtr.25920.1.S1_at | Hypothetical protein | Affymetrix |
| Mtr.21537.1.S1_at | Hypothetical protein | Affymetrix |
| Mtr.27335.1.S1_at | F2J6.14 protein | Affymetrix |
| Mtr.28358.1.S1_at | Protein phosphatase 2C-like | BLAST |
| Mtr.35218.1.S1_at | Unknown |  |
| Mtr.35438.1.S1_at | Unknown |  |
| Mtr.41666.1.S1_at | Unknown |  |
| Mtr.41682.1.S1_at | F5D14.28 protein, ABC family protein | BLAST |
| Mtr.43744.1.S1_at | Carboxyl-terminal proteinase | BLAST |
| Mtr.44425.1.S1_at | Ribitol kinase; Pentulose kinase; Carbohydrate kinase | BLAST |
| Mtr.50084.1.S1_at | Hypothetical protein | Affymetrix |
| Mtr.49942.1.S1_at | Protein kinase | BLAST |
| Mtr.803.1.S1_s_at | PHO1-like protein; EXS family protein | BLAST |
| Mtr.9581.1.S1_at | G2484-1 protein | Affymetrix |
| Mtr.6255.1.S1_at | Unknown |  |
| AFFX-TrpnX-5_at | Anthranilate phosphoribosyltransferase | Affymetrix |
| AFFX-TrpnX-3_at | N-(5'-phosphoribosyl)anthranilate isomerase | Affymetrix |
| AFFX-r2-Bs-phe-5_at | PheB; Chorismate mutase | BLAST |
| Mtr.103.1.S1_at | Unknown |  |
| Mtr.24089.1.S1_at | Unknown |  |
| Mtr.25949.1.S1_at | Unknown |  |
| Mtr.28754.1.S1_at | Unknown |  |
| Mtr.27255.1.S1_at | Unknown |  |
| Mtr.3495.1.S1_at | Zinc finger (C3HC4-type RING finger) family protein | BLAST |
| Mtr.3358.1.S1_at | Unknown |  |
| Mtr.3453.1.S1_s_at | Unknown |  |
| Mtr.35312.1.S1_at | Unknown |  |
| Mtr.35860.1.S1_at | Translocon-associated protein alpha (TRAP alpha) family protein; Signal sequence receptor, alpha subunit (SSR-alpha) | BLAST |
| Mtr.11836.1.S1_at | F-box protein | BLAST |
| Mtr.50283.1.S1_at | Hypothetical protein | Affymetrix |
| Mtr.46459.1.S1_at | Unknown |  |
| Mtr.47078.1.S1_at | Unknown |  |
| Mtr.48025.1.S1_at | Unknown |  |
| Mtr.7991.1.S1_at | Unknown |  |
| Mtr.38696.1.S1_s_at | Pre-mRNA splicing factor PRP38 family protein | BLAST |
