# Supplementary material for: Comparative gene expression profiles between heterotic and non-heterotic hybrids of tetraploid Medicago sativa
Source: BMC Plant Biol. 2009 Aug 13;9:107. doi: 10.1186/1471-2229-9-107 (PMC2736959; doi:10.1186/1471-2229-9-107)
Supplement: Additional file 4 — Primers used for qRT-PCR confirmation of microarray results [file 1471-2229-9-107-S4.doc]

### Additional file 4 - Primers used for qRT-PCR confirmation of microarray results

| Probe sets | Sequence |
| --- | --- |
| MTR.10682F | ATT GTG GGT ATG CAG GGA AA |
| MTR.10682R | GAT GCA CGT TCA ACC AGA AA |
| MTR.11026F | GGA AGG TTC CTA TGC ACG AA |
| MTR.11026R | CAT TGC AAA ACC AGG ATC AA |
| MTR.18125F | GAA TTA CGA AGC CTG CGA TT |
| MTR.18125R | TTC TCC TAG CGT GCT CTG TG |
| MTR.241F | TAG GGT TGG CTC TGT TTT GG |
| MTR.241R | TCC ATG AGC TTG CTT CTC AA |
| MTR.3074F | TGT TCT CTC GAT CTG CAT GG |
| MTR.3074R | GCT CCC ACC CAG AGT CTG TA |
| MTR.34420F | GCT CGC TAA GAA TGG GTT TG |
| MTR.34420R | GCT TCA TCA AGA TGC CCA AT |
| MTR.37570F | TGT CAT GGC GGT TGA TAA GA |
| MTR.37570R | CAT CCC AAG CAC AGG AAA AA |
| MTR.9194F | AGA AGG GGG AAA CTT CTG GA |
| MTR.9194R | GCA GTA ACA CGC TCC CTC TC |
| MTR.43518F | ATC CAT GTT ACC CGT TTC TCA |
| MTR.43518R | TCA AAA AGT TTT GCC CTC CA |
